# Up-regulation of ACE2, the SARS-CoV-2 receptor, in asthmatics on maintenance inhaled corticosteroids

**DOI:** 10.1186/s12931-021-01782-0

**Published:** 2021-07-07

**Authors:** Sarah L. O’Beirne, Jacqueline Salit, Robert J. Kaner, Ronald G. Crystal, Yael Strulovici-Barel

**Affiliations:** 1 Department of Genetic Medicine, Weill Cornell Medical College, 1300 York Avenue, Box 164, New York, NY 10065 USA; 2 Department of Medicine, Weill Cornell Medical College, New York, NY USA

**Keywords:** Asthma, Maintenance inhaled corticosteroids, ACE2, SARS-CoV-2, Gene expression, Large airway epithelium

## Abstract

**Background:**

The first step in SARS-CoV-2 infection is binding of the virus to angiotensin converting enzyme 2 (ACE2) on the airway epithelium. Asthma affects over 300 million people world-wide, many of whom may encounter SARS-CoV-2. Epidemiologic data suggests that asthmatics who get infected may be at increased risk of more severe disease. Our objective was to assess whether maintenance inhaled corticosteroids (ICS), a major treatment for asthma, is associated with airway ACE2 expression in asthmatics.

**Methods:**

Large airway epithelium (LAE) of asthmatics treated with maintenance ICS (ICS^+^), asthmatics not treated with ICS (ICS^−^), and healthy controls (controls) was analyzed for expression of ACE2 and other coronavirus infection-related genes using microarrays.

**Results:**

As a group, there was no difference in LAE ACE2 expression in all asthmatics vs controls. In contrast, subgroup analysis demonstrated that LAE ACE2 expression was higher in asthmatics ICS^+^ compared to ICS‾ and ACE2 expression was higher in male ICS^+^ compared to female ICS^+^ and ICS‾ of either sex. ACE2 expression did not correlate with serum IgE, absolute eosinophil level, or change in FEV1 in response to bronchodilators in either ICS^−^ or ICS^+^.

**Conclusion:**

Airway ACE2 expression is increased in asthmatics on long-term treatment with ICS, an observation that should be taken into consideration when assessing the use of inhaled corticosteroids during the pandemic.

**Supplementary Information:**

The online version contains supplementary material available at 10.1186/s12931-021-01782-0.

## Introduction

The global pandemic coronavirus disease-2019 (COVID-19), caused by infection with severe acute respiratory syndrome coronavirus 2 (SARS-CoV-2), is characterized by high morbidity and mortality, predominantly from respiratory failure [1]. Moderate to severe asthma may be associated with an increased risk of being infected with COVID-19 [2, 3]. Possible explanations include differences in patient behavior, the underlying disease process, and/or the medications used to treat the disease [4]. Inhaled corticosteroids (ICS) are the cornerstone of asthma therapy, reducing airway inflammation, controlling asthma symptoms and preventing exacerbations [5]. The immunosuppressive properties of inhaled and systemic corticosteroids have led to concerns about their use during the pandemic. However, without clear evidence of detriment, current respiratory society guidelines recommend their continued therapy to maintain disease control and to use systemic corticosteroids during exacerbations, if required [2, 4, 6, 7]. In the context that inhaled corticosteroids are commonly used to treat asthma, and that dexamethasone is widely used to treat COVID-19 related pneumonia, we asked: are there COVID-19-related risks to the use of corticosteroids in patients with asthma?

SARS-CoV-2 uses the angiotensin converting enzyme 2 (ACE2) receptor to enter host cells [8]. We previously demonstrated that the ACE2 gene is widely expressed in the human airway epithelium and is upregulated in males vs females and smokers vs nonsmokers [9]. Several studies examined ACE2 expression in asthmatics, including in the nasal, large airway epithelium (LAE) and lung biopsies [10–15]. However, none of these studies directly addressed the effects of chronic use of ICS on airway ACE2 expression in asthmatics. Given that asthma affects over 300 million people, many of whom are on treatment with inhaled corticosteroids and may encounter SARS-CoV-2, we evaluated LAE ACE2 expression in a cohort of predominantly mild to moderate nonsmoker asthmatics, treated with maintenance ICS (ICS^+^) vs not treated (ICS^−^).

## Methods

### Study population and biologic samples

Research subjects were recruited using local print and online media and evaluated at the Weill Cornell Medical College Clinical Translational and Science Center and the Department of Genetic Medicine Clinical Research Facility under institutional review board-approved protocols. After providing written consent, all subjects underwent a detailed screening visit, including assessment of medical history, physical exam, complete blood count, coagulation studies, liver function tests, urine analysis, chest X-ray, high resolution chest CT scan, EKG and pulmonary function tests to determine their phenotype 1–3 weeks prior to gene expression assessment. All subjects were determined to be nonsmokers based on self-reported smoking history, confirmed by the absence of the tobacco metabolites nicotine and cotinine in their urine [16] (see Inclusion/Exclusion criteria). Asthma was diagnosed based on a compatible clinical history plus evidence of reversible airflow obstruction and/or positive methacholine challenge [17]. The study population included nonsmokers with asthma (n = 57, “all asthma”) and healthy nonsmoker controls (n = 29, “controls”), see Table 1 for demographic details. Of the n = 57 asthmatics, n = 19 reported to be on maintenance inhaled corticosteroids (“ICS”) twice a day, with doses ranging from 50 to 250 mcg (average dose 186 mcg), for at least 3 weeks to 6.5 years (average use at least 6 months, “ICS^+”^), and n = 38 were not treated (“ICS^−“^).

**Table 1:** Study population demographics

Parameters	Controls	Asthmatics			p value	
		All	ICS‾	ICS^+^	All asthmatics vs controls	Asthmatics ICS^−^ vs ICS^+^
N	29	57	38	19		
Sex (male/female)	11/18	30/27	25/13	5/14	> 0.1	< 0.005
Age	38 ± 11	33 ± 11	31 ± 10	36 ± 11	< 0.04	> 0.08
Ethnicity (AA/E/H/O)^1^	13/8/4/4	31/10/13/3	24/5/6/3	7/5/7/0	> 0.5	> 0.2
BMI (kg/m^2^)	26 ± 4	27 ± 4	27 ± 5	27 ± 4	> 0.09	> 0.9
IgE (IU/mL)	38 ± 37	471 ± 674	509 ± 774	394 ± 417	< 0.002	> 0.5
Eosinophils (absolute, × 10^3^/uL)	0.1 ± 0.1	0.3 ± 0.2	0.3 ± 0.2	0.3 ± 0.2	< 10^–4^	> 0.9
Lung function^2^						
FVC (% predicted)	112 ± 12	102 ± 14	103 ± 13	101 ± 18	< 0.002	> 0.7
FEV_1_ (% predicted)	109 ± 11	87 ± 13	88 ± 13	84 ± 13	< 10^–11^	> 0.3
FEV1 response to ronchodilators (% change)	8 ± 2	11 ± 8	11 ± 8	12 ± 10	< 0.006	> 0.5
FEV_1_/FVC (% observed)	81 ± 4	71 ± 9	72 ± 8	70 ± 9	< 10^–6^	> 0.4
TLC (% predicted)	102 ± 16	103 ± 15	103 ± 15	103 ± 16	> 0.7	> 0.8
DLCO (% predicted)	90 ± 8	89 ± 11	89 ± 12	88 ± 10	> 0.6	> 0.5
Asthma severity (mild/mod/sev)^3^	N/A	29/23/5	25/10/3	4/13/2	N/A	< 0.02
LAE asthma-related genes^4^						
CLCA1	2 ± 11	27 ± 59	26 ± 45	30 ± 82	< 10^–6^ (12.0)	> 0.1
SERPINB2	5 ± 5	30 ± 36	32 ± 34	27 ± 41	< 10^–6^ (3.9)	> 0.3
POSTN	16 ± 15	63 ± 66	74 ± 73	40 ± 41	< 10^–5^ (3.2)	< 0.03 (2.0)

Data is presented as mean ± standard deviation; p value calculated using a Student’s t-test for numerical parameters or a chi-square test for categorical parameters; all subjects were HIV negative, nonsmokers, and had normal levels of α1-antitrypsin (see Inclusion/Exclusion criteria in Additional file 1 for details); controls = healthy nonsmoker controls, ICS^−^ = nonsmoker asthmatics not treated with inhaled corticosteroids (ICS), ICS^+^  = nonsmoker asthmatics treated with maintenance ICS

^1^*AA* African-American; *E* European; *H* Hispanic; *O* Other

^2^Lung function parameters are presented as pre-bronchodilator values and as percent predicted except the FEV_1_/FVC ratio, which is presented as % observed; FEV1 response to bronchodilators was calculated based on FEV1 post vs pre-bronchodilators (% change); *FVC* forced vital capacity; *FEV*_1_ forced expiratory volume in 1 s; *TLC* total lung capacity; *DLCO* diffusion capacity, DLCO was corrected for hemoglobin and carboxyhemoglobin [35]

^3^*Mod* moderate, *sev* severe, *N/A*  not applicable

^4^Large airway epithelium (LAE) gene expression was assessed by microarray (Affymetrix HG-U133 Plus 2.0) for genes expressed in ≥ 20% of the subjects in either compared group; additional LAE asthma-related genes [24]; IL-4, IL-5 and IL-13 were expressed in < 20% of the subjects, therefore were not compared between the groups; all asthmatics vs controls were compared using 2-way ANCOVA (age was a co-variant), significant genes were up-regulated in asthmatics vs controls; asthmatics ICS^−^ vs ICS^+^ were compared using 2-way ANCOVA (sex was a co-variant), significant genes were up-regulated in ICS^**−**^ vs ICS^**+**^; the fold-change is detailed in brackets for genes with a significant p value (p value < 0.05 was considered significant)

### Sampling of the large airway epithelium

After undergoing evaluation, all individuals who met the Inclusion/Exclusion criteria listed above underwent bronchoscopy with brushing of the large airway to sample the epithelium as previously described [9, 18]. Briefly, a fiberoptic bronchoscope was positioned proximal to the opening of a desired lobar bronchus and a 2 mm diameter brush was used for gentle brushing of the 3rd–4th order bronchi. Cells were collected by gently gliding the brush back and forth on the epithelium 5–10 times in 8–10 different locations in the same general area.

### Large airway epithelium sample processing

LAE cells were processed as previously described [9, 18]. Briefly, LAE cells were dislodged from the cytology brush by flicking into 5 ml of ice-cold Bronchial Epithelium Basal Medium (BEBM, Lonza, Basel, Switzerland) and kept on ice until processed. A 4.5 ml aliquot was immediately processed for RNA extraction and the remaining 0.5 ml aliquot was used for differential cell count. Total cell number was determined by counting on a hemocytometer and cell morphology and differential cell count (percentage of inflammatory and epithelial cells as well as proportions of ciliated, basal, secretory, and undifferentiated epithelial cells) were assessed on sedimented cells prepared by centrifugation (Cytospin 11, Shandon instruments, Pittsburgh, PA) and stained with Diff-Quik (Dade Behring, Newark, NJ).

### Microarray assessment of gene expression

Total RNA was prepared for microarray transcriptome analysis using the 3’IVT Express kit (Affymetrix, Santa Clara, CA) and assessed using Affymetrix HG-U133 Plus 2.0 microarrays (Affymetrix), as previously described [19, 20]. Briefly, RNA quantity was assessed by Nanodrop ND-1000 (Thermo Scientific, Wilmington, DE) and RNA quality by Bioanalyzer (Agilent Technologies, Santa Clara, CA) [21, 22]. Total RNA (1–2 µg) was used to synthesize double stranded cDNA and Affymetrix kits were used to quantify the biotin-labeled cDNA yield^5^. RNA was hybridized on the arrays with probes for > 54,000 genome-wide transcripts using Affymetrix protocols, hardware and software [22]. Microarray quality was verified by signal intensity ratio of GAPDH 3' to 5' probe sets ≤ 3.0 and multi-chip normalization scaling factor ≤ 10.0 [21]. The MAS5 algorithm (GeneSpring version 7.3, Affymetrix Microarray Suite Version 5) was used to normalize the data per array to the median expression value of each sample. Each gene was represented by one probeset, selected based on highest specificity and sensitivity scores (Affymetrix) and its expression level was assessed relatively to the gene expression of all other genes on the array (n = 14,465). Assessment of gene expression was performed for genes with relative expression level > 0 in at least ≥ 20% of the subjects in either one of the compared groups. In addition to ACE2, the expression of other genes related to the initial steps of coronavirus and several LAE asthma-related genes was compared between the groups. The image files from the microarrays were processed using Partek Genomics Suite software version 6.6, 2012 (Partek, St. Louis, MO). A 2-way ANCOVA was used to compare all asthmatics vs controls (age was a co-variant) and asthmatics ICS^+^ vs ICS^−^ (sex was a co-variant) in Partek (p < 0.05 was considered significant). The raw data analyzed in this study has been deposited in the public repository Gene Expression Omnibus (www.ncbi/nlm/nih.gov/geo, accession # 179156).

## Results

Current epidemiologic data suggests that asthmatics who are infected with SARS-CoV-2 may be at increased risk of more severe disease [2, 3]. Airway epithelial expression of ACE2 plays a central role in SARS-CoV-2 infection [7] and its expression in the asthmatic airway may be influenced by T-helper type 2 (Th2)-related inflammatory processes [13, 23]. Therapies such as inhaled corticosteroids may be relevant to this observation. Consistent with this hypothesis, we observed higher large airway epithelial ACE2 expression in asthmatics ICS^+^ vs ICS^−^.

With minor differences, assessment of demographics of all asthmatics, as a group, and controls demonstrated the groups were similar in sex, ethnicity and BMI (Table 1). For gene expression comparison of the groups, the difference in age was accounted for using ANCOVA. ICS^+^ and ICS^−^ asthmatics were comparable in all demographics, except for sex, which was accounted for in the gene expression analysis using ANCOVA. As expected, the asthmatics, as a group, had higher IgE and eosinophil levels compared to controls, but there was no difference in these parameters among asthmatics ICS^+^ vs ICS^−^. As demonstrated by others [24], quantification of expression of the LAE asthma-related genes CLCA1, SERPINB2 and POSTN confirmed they were up-regulated in asthmatics vs controls. Of those, only POSTN was up-regulated in asthmatics ICS^−^ vs ICS^+^. IL-4, 5 and 13 were expressed in < 20% of the subjects and therefore not compared between the groups.

**Fig. 1:**
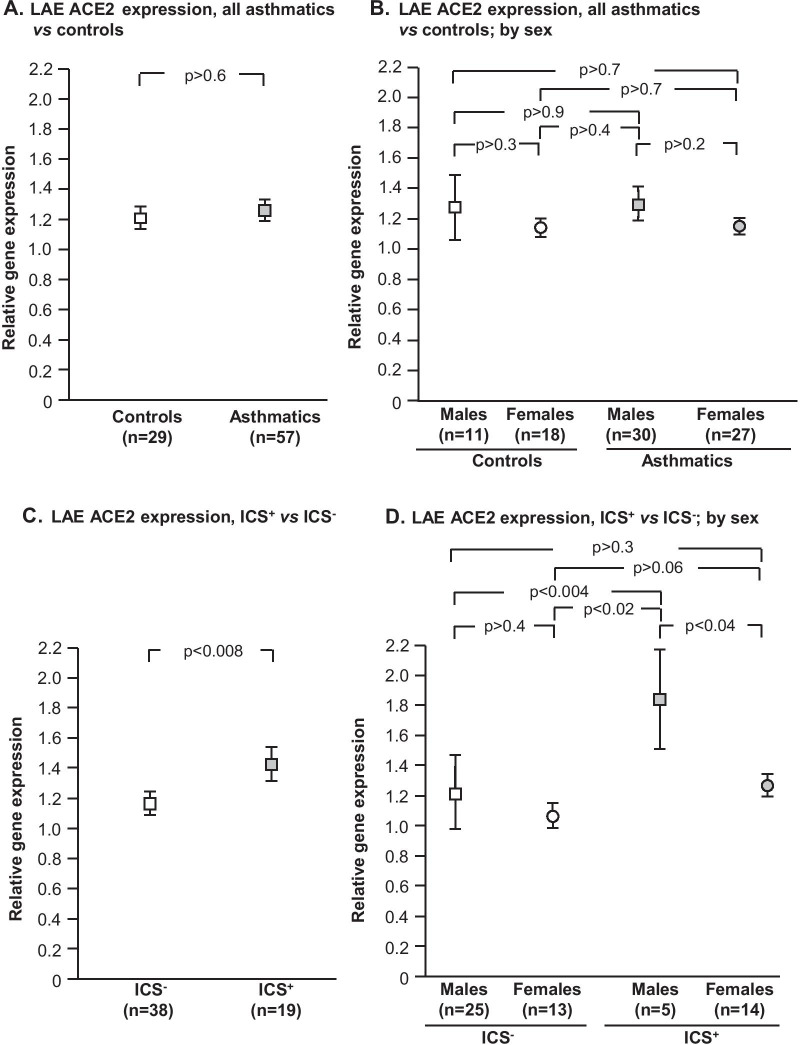
Effect of maintenance inhaled corticosteroid (ICS) and sex on ACE2 expression in the large airway epithelium (LAE). **A**, **B** All nonsmoker asthmatics (n = 57) vs healthy nonsmoker controls (n = 29). **C**, **D** Nonsmoker asthmatics on maintenance ICS (ICS^+^, n = 19) vs nonsmoker asthmatics not on ICS (ICS^−^, n = 38). **A** All asthmatics vs controls, by phenotype (ANCOVA, with age as a co-variant). **B** All asthmatics vs controls, by phenotype and sex (ANOVA). **C** Asthmatics ICS^+^ vs asthmatics ICS^−^, by phenotype (ANCOVA, with sex as a co-variant). **D** Asthmatics ICS^+^ vs asthmatics ICS^−^, by phenotype and sex (ANOVA). Data is presented as mean ± standard error

**Fig. 2:**
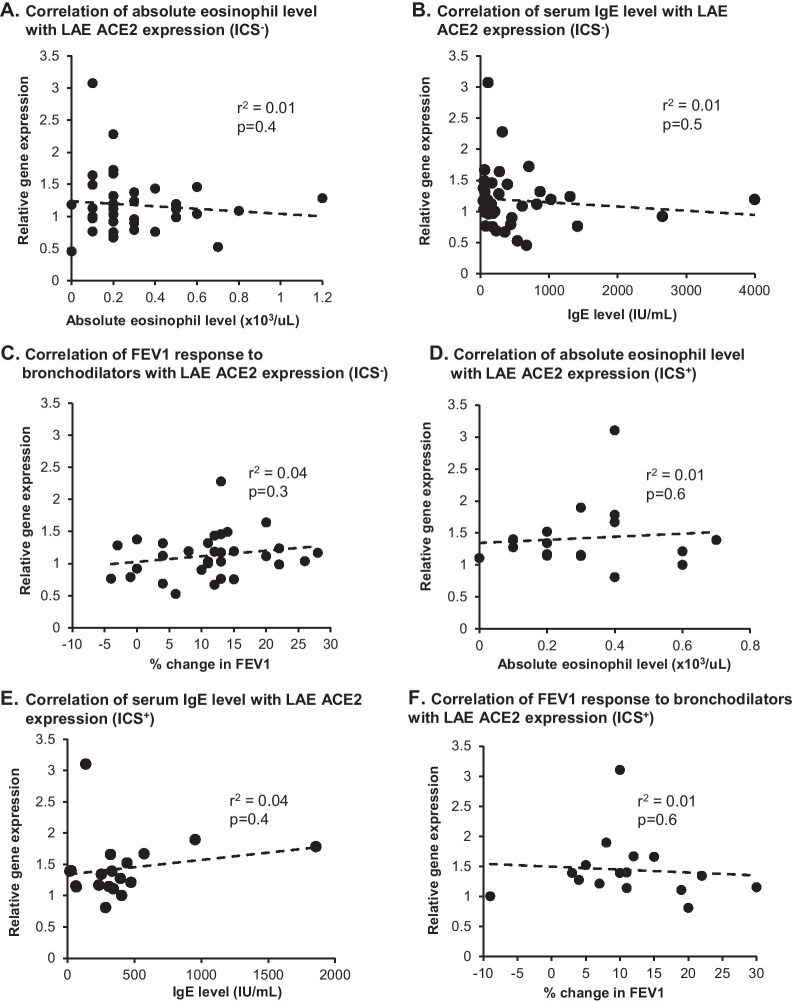
Correlation of absolute eosinophil and serum IgE levels and FEV1 response to bronchodilators with large airway epithelium (LAE) ACE2 expression. **A–C** Nonsmoker asthmatics not treated with inhaled corticosteroids (ICS, ICS^−^, n = 38). **D–F** Nonsmoker asthmatics treated with maintenance ICS (ICS^+^, n = 19). **A**, **D** Eosinophil level vs ACE2 expression. **B**, **E** IgE level vs ACE2 expression. **C**, **F** FEV1 response to bronchodilators (FEV1 post- vs pre-bronchodilators, presented as % change) vs ACE2 expression

There was no difference in the LAE ACE2 expression in all asthmatics, as a group compared to controls (all subjects, p > 0.2; analyzed by sex, p > 0.2, Fig. 1A, B). However, ACE2 expression was higher in the LAE of asthmatics ICS^+^ vs asthmatics ICS^−^ (p < 0.008, fold-change > 1.2, Fig. 1C). Consistent with our prior observation of higher ACE2 expression in males [9], male asthmatics treated with ICS exhibited significantly higher ACE2 expression than female ICS^+^ (p < 0.04, fold-change > 1.5), male ICS^−^ (p < 0.004, fold-change > 1.5) and female ICS- (p < 0.02, fold-change > 1.7, all comparisons, Fig. 1D). ACE2 expression did not correlate with serum IgE or absolute eosinophil levels, or with change in FEV1 in response to bronchodilator in either ICS^+^ or ICS^−^ (r^2^ < 0.05 and p > 0.2, all correlations, Fig. 2).

In contrast to our observation that chronic use of inhaled corticosteroids up-regulates ACE2 expression in the LAE, based on our analysis of the data set of Woodruff et al. [24] (www.ncbi.nlm.nih.gov/geo, accession #4302) of LAE gene expression before and after acute exposure to inhaled fluticasone for 1 week, ACE2 expression was not up-regulated in asthmatics in response to the treatment. We conclude that acute exposure to inhaled corticosteroids has no effect on LAE ACE2 expression, while chronic use of inhaled corticosteroids is associated with LAE up-regulation of ACE2.

In addition to assessing the expression of ACE2, we assessed the LAE expression of other genes related to the initial steps of coronavirus infection based on lineage B coronaviruses, including: ADAM10 and ADAM17 (ADAM metallopeptidase domain 10 and 17, respectively), cell surface disintegrins that mediate shedding of ACE2 from the cell surface [25]; TMPRSS2, TMPRSS11A and TMPRS11D (transmembrane protease serine 2, 11A and 11D, respectively), CSTL (cathepsin L) and FURIN (furin), involved in protein priming of the virus to facilitate fusion of the coronavirus envelope with the cell membrane during infection by SARS-CoV-2 [8, 26–28]; and PIK4B (phosphatidylinositol 4-kinase beta), an enzyme that inhibits SARS-CoV-2 infection [29]. All genes, except for FURIN, were expressed in the LAE. Similar to our observations in smokers compared to nonsmokers [9], other than the LAE up-regulation of TMPRSS2 in asthmatics overall compared to controls (p < 0.004, fold-change = 1.2), there were no other asthma-related changes in LAE expression among the genes related to coronavirus infection (p > 0.1, all comparisons). In regard to ICS^+^ vs ICS^−^, up-regulation of TMPRSS11D was the only ICS-related change in LAE expression, with higher expression among ICS^−^ compared to ICS^+^ (p < 0.05, fold-change = 1.3).

## Discussion

Our observation of induced LAE ACE2 expression of asthmatics on maintenance inhaled corticosteroids vs asthmatics not on corticosteroids needs to be put in the context of other observations of ACE2 expression in asthmatics. Several studies that examined ACE2 expression in asthmatics found no significant difference between asthmatics and controls, and no relationship between ACE2 expression and asthma severity [11, 14]. A small decrease in ACE2 expression in nasal epithelium samples has been noticed in asthmatics compared with healthy controls [13]. ACE2 expression in sputum cells was similar in asthmatics vs controls, but lower in asthmatics on ICS vs asthmatics not on ICS [30]. Finally, a study of ACE2 expression in nasal airway samples obtained from children and adults with allergic rhinitis and asthma reported an inverse relationship between nasal ACE2 expression and allergic status and Th2 biomarkers, including serum IgE, exhaled nitric oxide level, and reduced nasal and airway IL-13 expression and airway ACE2 expression following allergen exposure [10]. This clinical observation is supported by in vitro studies reporting a reduction in ACE2 expression by airway epithelial cells following IL-13 treatment and a negative correlation between ACE2 and Th2-cytokines [25].

New data on the effects of asthma on SARS-CoV-2 infection rate and disease severity are rapidly evolving. Several studies found no association of asthma with an increased risk of hospitalization or duration of hospitalization due to COVID-19 [31, 32], while others found that asthma patients likely have different risk of severe COVID-19, which may be associated with different ACE2 expression [33]. However, most studies agree that the variability in asthma prevalence among patients with COVID-19 in different countries or regions makes it difficult to draw strong conclusions regarding the risk, if any, of asthmatics to infection with SARS-CoV-2 [33, 34]. This study demonstrated that ACE2 expression is significantly higher in ICS^+^ compared to ICS^−^ asthmatics, but to a small extent, possibly due to the limitation in size of the study population. A study that compares the rate of SARS-CoV-2 infection in ICS^+^ vs ICS^−^ asthmatics, with larger sample sizes is required to verify our findings.

## Conclusions

Asthmatics on maintenance inhaled corticosteroids have higher expression of LAE ACE2 than those not on corticosteroids. It is not clear whether this is secondary to the corticosteroids per se or related to processes associated with the underlying asthma. In the context that increased LAE ACE2 expression may increase susceptibility to the extent of infection in asthmatics exposed to SARS-CoV-2, although ICS are the cornerstone of asthma therapy, and asthma should be controlled in all patients, management of patients with asthma infected by SARS-CoV-2, including adjustment of medication, should be considered on an individual basis.

## Supplementary Information

**Additional file1.** Inclusion and exclusion criteria.

## Data Availability

The raw data analyzed in this study has been deposited in the public repository Gene Expression Omnibus (www.ncbi/nlm/nih.gov/geo, accession # 179156).

## Abbreviations

ACE2: Angiotensin converting enzyme 2
ANCOVA: Analysis of covariance
Controls: Nonsmoker healthy controls
COVID-19: Coronavirus disease-2019
FEV1: Forced expiatory volume in one second
ICS: Inhaled corticosteroids
ICS^+^: Nonsmoker asthmatics, treated with maintenance ICS
ICS^−^: Nonsmoker asthmatics, not treated with ICS
LAE: Large airway epithelium
SARS-CoV-2: Severe acute respiratory syndrome coronavirus 2

## References

[1] Guan WJ, Liang WH, Zhao Y, Liang HR, Chen ZS, Li YM, Liu XQ, Chen RC, Tang CL, Wang T (2020). Comorbidity and its impact on 1590 patients with COVID-19 in China: a nationwide analysis. Eur Respir J.

[2] Hartmann-Boyce J, Gunnell J, Drake J, Otunla A, Suklan J, Schofield E, Kinton J, Inada-Kim M, Hobbs FDR, Dennison P (2020). Asthma and COVID-19: review of evidence on risks and management considerations. BMJ Evid Based Med.

[3] Coronavirus (COVID-19), Centers for Disease Control and Prevention. 2020. https://www.cdc.gov/coronavirus. Accessed 7 June 2021.

[4] Halpin DMG, Faner R, Sibila O, Badia JR, Agusti A (2020). Do chronic respiratory diseases or their treatment affect the risk of SARS-CoV-2 infection?. Lancet Respir Med.

[5] GINA: Interim Guidance About COVID-19 & Asthma - Updated 26 April 2021; Global Initiative for Asthma. https://ginasthma.org/. Accessed 7 June 2021.

[6] Morais-Almeida M, Aguiar R, Martin B, Ansotegui IJ, Ebisawa M, Arruda LK, Caminati M, Canonica GW, Carr T, Chupp G (2020). COVID-19, asthma, and biological therapies: what we need to know. World Allergy Organ J.

[7] Horby P, Lim WS, Emberson JR, Mafham M, Bell JL, Linsell L, Staplin N, Brightling C, Ustianowski A, Group RC (2020). Dexamethasone in hospitalized patients with Covid-19—preliminary report. N Engl J Med.

[8] Hoffmann M, Kleine-Weber H, Schroeder S, Kruger N, Herrler T, Erichsen S, Schiergens TS, Herrler G, Wu NH, Nitsche A (2020). SARS-CoV-2 cell entry depends on ACE2 and TMPRSS2 and is blocked by a clinically proven protease inhibitor. Cell.

[9] Zhang H, Rostami MR, Leopold PL, Mezey JG, O'Beirne SL, Strulovici-Barel Y, Crystal RG (2020). Expression of the SARS-CoV-2 ACE2 receptor in the human airway epithelium. Am J Respir Crit Care Med.

[10] Jackson DJ, Busse WW, Bacharier LB, Kattan M, O'Connor GT, Wood RA, Visness CM, Durham SR, Larson D, Esnault S (2020). Association of respiratory allergy, asthma, and expression of the SARS-CoV-2 receptor ACE2. J Allergy Clin Immunol.

[11] Bradding P, Richardson M, Hinks TSC, Howarth PH, Choy DF, Arron JR, Wenzel SE, Siddiqui S (2020). ACE2, TMPRSS2, and furin gene expression in the airways of people with asthma-implications for COVID-19. J Allergy Clin Immunol.

[12] Camiolo M, Gauthier M, Kaminski N, Ray A, Wenzel SE (2020). Expression of SARS-CoV-2 receptor ACE2 and coincident host response signature varies by asthma inflammatory phenotype. J Allergy Clin Immunol.

[13] Kimura H, Francisco D, Conway M, Martinez FD, Vercelli D, Polverino F, Billheimer D, Kraft M (2020). Type 2 inflammation modulates ACE2 and TMPRSS2 in airway epithelial cells. J Allergy Clin Immunol.

[14] Radzikowska U, Ding M, Tan G, Zhakparov D, Peng Y, Wawrzyniak P, Wang M, Li S, Morita H, Altunbulakli C (2020). Distribution of ACE2, CD147, CD26, and other SARS-CoV-2 associated molecules in tissues and immune cells in health and in asthma, COPD, obesity, hypertension, and COVID-19 risk factors. Allergy.

[15] Yao Y, Wang H, Liu Z (2020). Expression of ACE2 in airways: implication for COVID-19 risk and disease management in patients with chronic inflammatory respiratory diseases. Clin Exp Allergy.

[16] Moyer TP, Charlson JR, Enger RJ, Dale LC, Ebbert JO, Schroeder DR, Hurt RD (2002). Simultaneous analysis of nicotine, nicotine metabolites, and tobacco alkaloids in serum or urine by tandem mass spectrometry, with clinically relevant metabolic profiles. Clin Chem.

[17] Crapo RO, Casaburi R, Coates AL, Enright PL, Hankinson JL, Irvin CG, MacIntyre NR, McKay RT, Wanger JS, Anderson SD (2000). Guidelines for methacholine and exercise challenge testing-1999. This official statement of the American Thoracic Society was adopted by the ATS Board of Directors, July 1999. Am J Respir Crit Care Med.

[18] Vanni H, Kazeros A, Wang R, Harvey BG, Ferris B, De BP, Carolan BJ, Hubner RH, O'Connor TP, Crystal RG (2009). Cigarette smoking induces overexpression of a fat-depleting gene AZGP1 in the human. Chest.

[19] Strulovici-Barel Y, Omberg L, O'Mahony M, Gordon C, Hollmann C, Tilley AE, Salit J, Mezey J, Harvey BG, Crystal RG (2010). Threshold of biologic responses of the small airway epithelium to low levels of tobacco smoke. Am J Respir Crit Care Med.

[20] Tilley AE, O'Connor TP, Hackett NR, Strulovici-Barel Y, Salit J, Amoroso N, Zhou XK, Raman T, Omberg L, Clark A (2011). Biologic phenotyping of the human small airway epithelial response to cigarette smoking. PLoS ONE.

[21] Raman T, O'Connor TP, Hackett NR, Wang W, Harvey BG, Attiyeh MA, Dang DT, Teater M, Crystal RG (2009). Quality control in microarray assessment of gene expression in human airway epithelium. BMC Genomics.

[22] Tumor Analysis Best Practices Working G. Expression profiling—best practices for data generation and interpretation in clinical trials. Nat Rev Genet. 2004;5:229–37. 10.1038/nrg1297 14970825

[23] Ziegler CGK, Allon SJ, Nyquist SK, Mbano IM, Miao VN, Tzouanas CN, Cao Y, Yousif AS, Bals J, Hauser BM (2020). SARS-CoV-2 receptor ACE2 is an interferon-stimulated gene in human airway epithelial cells and is detected in specific cell subsets across tissues. Cell.

[24] Woodruff PG, Modrek B, Choy DF, Jia G, Abbas AR, Ellwanger A, Koth LL, Arron JR, Fahy JV (2009). T-helper type 2-driven inflammation defines major subphenotypes of asthma. Am J Respir Crit Care Med.

[25] Jia HP, Look DC, Tan P, Shi L, Hickey M, Gakhar L, Chappell MC, Wohlford-Lenane C, McCray PB (2009). Ectodomain shedding of angiotensin converting enzyme 2 in human airway epithelia. Am J Physiol Lung Cell Mol Physiol.

[26] Shulla A, Heald-Sargent T, Subramanya G, Zhao J, Perlman S, Gallagher T (2011). A transmembrane serine protease is linked to the severe acute respiratory syndrome coronavirus receptor and activates virus entry. J Virol.

[27] Bosch BJ, Bartelink W, Rottier PJ (2008). Cathepsin L functionally cleaves the severe acute respiratory syndrome coronavirus class I fusion protein upstream of rather than adjacent to the fusion peptide. J Virol.

[28] Simmons G, Zmora P, Gierer S, Heurich A, Pohlmann S (2013). Proteolytic activation of the SARS-coronavirus spike protein: cutting enzymes at the cutting edge of antiviral research. Antiviral Res.

[29] Yang N, Ma P, Lang J, Zhang Y, Deng J, Ju X, Zhang G, Jiang C (2012). Phosphatidylinositol 4-kinase IIIbeta is required for severe acute respiratory syndrome coronavirus spike-mediated cell entry. J Biol Chem.

[30] Peters MC, Sajuthi S, Deford P, Christenson S, Rios CL, Montgomery MT, Woodruff PG, Mauger DT, Erzurum SC, Johansson MW (2020). COVID-19-related genes in sputum cells in asthma. Relationship to demographic features and corticosteroids. Am J Respir Crit Care Med.

[31] Chhiba KD, Patel GB, Vu THT, Chen MM, Guo A, Kudlaty E, Mai Q, Yeh C, Muhammad LN, Harris KE (2020). Prevalence and characterization of asthma in hospitalized and nonhospitalized patients with COVID-19. J Allergy Clin Immunol.

[32] Wang Y, Chen J, Chen W, Liu L, Dong M, Ji J, Hu D, Zhang N (2021). Does asthma increase the mortality of patients with COVID-19?: A systematic review and meta-analysis. Int Arch Allergy Immunol.

[33] Song J, Zeng M, Wang H, Qin C, Hou HY, Sun ZY, Xu SP, Wang GP, Guo CL, Deng YK (2021). Distinct effects of asthma and COPD comorbidity on disease expression and outcome in patients with COVID-19. Allergy.

[34] Timberlake DT, Strothman K, Grayson MH (2021). Asthma, severe acute respiratory syndrome coronavirus-2 and coronavirus disease 2019. Curr Opin Allergy Clin Immunol.

[35] Macintyre N, Crapo RO, Viegi G, Johnson DC, van der Grinten CP, Brusasco V, Burgos F, Casaburi R, Coates A, Enright P (2005). Standardisation of the single-breath determination of carbon monoxide uptake in the lung. Eur Respir J.

