# Supplementary material for: Up-regulation of ACE2, the SARS-CoV-2 receptor, in asthmatics on maintenance inhaled corticosteroids
Source: Respir Res. 2021 Jul 7;22:200. doi: 10.1186/s12931-021-01782-0 (PMC8261394; doi:10.1186/s12931-021-01782-0)
Supplement: Supplementary file 1 — Additional file1. Inclusion and exclusion criteria. [file 12931_2021_1782_MOESM1_ESM.docx]

**Additional Methods**

**Inclusion and Exclusion Criteria**

**Asthmatic subjects: Inclusion criteria**

- Males and females, at least 18 years old
- Provide informed consent
- Normal physical examination
- HIV negative
- Not pregnant (females)
- Nonsmokers by history, with current smoking status validated by the following metabolites findings: urine nicotine <2 ng/ml and urine cotinine <5 ng/ml^1^
- History of allergic symptoms and at least one of the following: elevated IgE levels (>165.3 IU/ml), elevated eosinophil levels (≥0.45 x10^3^/µL)
- Evidence of reversible airflow obstruction on pulmonary function testing and/or positive methacholine challenge performed as per ATS guidelines^2^
- Normal PA and lateral chest radiograph
- Normal electrocardiogram
- Normal serum α1-antitrypsin levels
- Good health without history of chronic lung disease, excluding asthma, and without recurrent or recent (within 3 months) acute pulmonary disease
- No history of allergies to medications used during bronchoscopy

**Healthy controls: Inclusion criteria**

- Males and females, at least 18 years old
- Provide informed consent
- Good health without history of chronic lung disease, including asthma, and without recurrent or recent (within 3 months) acute pulmonary disease
- Normal physical examination
- HIV negative
- Not pregnant (females)
- Nonsmokers by history, with current smoking status validated by the following metabolites findings: urine nicotine <2 ng/ml and urine cotinine <5 ng/ml^1^
- Normal routine laboratory evaluation, including general hematologic studies, general serologic/immunologic studies, general biochemical analyses and urine analysis
- No history of allergic symptoms
- Normal IgE and eosinophil levels
- Normal lung function, including forced expiratory volume in 1 second (FEV1) ≥80% predicted, forced vital capacity (FVC) ≥80% predicted, FEV1/FVC (≥0.7) based on pre-bronchodilator spirometry and total lung capacity (TLC) ≥90% predicted and DLCO ≥80% predicted
- Normal posterior-anterior (PA) and lateral chest radiograph
- Normal electrocardiogram
- No history of allergies to medications used during bronchoscopy
- Not taking any medications relevant to lung disease or known to affect the airway epithelium
- Willingness to participate in the study

**All Subjects: Exclusion criteria**

- Unable to meet the inclusion criteria
- Current active infection or acute illness of any kind
- Evidence of malignancy within the past 5 years
- Alcohol or drug abuse within the past 6 months

**Additional References**

1. Moyer TP, Charlson JR, Enger RJ, Dale LC, Ebbert JO, Schroeder DR, et al. Simultaneous analysis of nicotine, nicotine metabolites, and tobacco alkaloids in serum or urine by tandem mass spectrometry, with clinically relevant metabolic profiles. Clin Chem 2002; 48:1460-71.

2. Crapo RO, Casaburi R, Coates AL, Enright PL, Hankinson JL, Irvin CG, et al. Guidelines for methacholine and exercise challenge testing-1999. This official statement of the American Thoracic Society was adopted by the ATS Board of Directors, July 1999. Am J Respir Crit Care Med 2000; 161:309-29.
